# Macrosomia is a risk factor for incident maternal chronic kidney disease

**DOI:** 10.1186/s12884-021-03695-8

**Published:** 2021-03-16

**Authors:** Mohammad Vahidi, Samaneh Asgari, Maryam Tohidi, Fereidoun Azizi, Farzad Hadaegh

**Affiliations:** 1 Prevention of Metabolic Disorders Research Center, Research Institute for Endocrine Sciences Shahid Beheshti University of Medical Sciences, Tehran, Iran; 2 Endocrine Research Center, Research Institute for Endocrine Sciences, Shahid Beheshti University of Medical Sciences, Tehran, Iran

**Keywords:** Chronic kidney disease, Gestational diabetes mellitus, Macrosomia

## Abstract

**Background:**

Gestational diabetes mellitus (GDM) and macrosomia are associated with several adverse outcomes including diabetes mellitus and cardiovascular diseases, however, the relationship between GDM/macrosomia with incident chronic kidney disease (CKD) is a matter of debate. The purpose of this study was to examine the association between the history of macrosomia with or without GDM and incident maternal CKD.

**Methods:**

The study population includes 2669 women aged 18–50 years without known diabetes mellitus and CKD from participants of the Tehran Lipid and Glucose Study. The study population was categorized into 3 groups; group 1: GDM/macrosomia and without diabetes mellitus (*n* = 204), group 2: newly diagnosed incident diabetes mellitus (NDM) in the presence or abcence of GDM/Macrosomia (*n* = 113), and, group 3: the reference group including women without prior history of GDM/macrosomia and free of NDM (*n* = 2352). CKD was defined as an estimated glomerular filtration rate (eGFR) < 60 ml/min/1.73 m^2^. Multivariable Cox proportional hazard regression adjusted for baseline values of age, body mass index, waist circumference, parity numbers, smoking, educational level, gestational hypertension, eGFR, systolic and diastolic blood pressures (SBP and DBP, respectively), anti-hypertensive medication, and family history of diabetes mellitus was applied for data analyses.

**Results:**

During a median follow-up of 11.9 years, 613 incident CKD cases were identified. The multivariable hazard ratio (HR) and 95% confidence interval (CI) on GDM/macrosomia group was [1.32 (1.02–1.72)]; the risk was more prominent among non-hypertensive women [1.41 (1.07–1.85); *P* for interaction: 0.046]. Moreover, the history of macrosomia alone also showed a significant risk [1.36 (1.04–1.78)]; however, history of GDM alone did not have a significant risk [0.92 (0.34–2.46)]. Age, current smoking, eGFR, and SBP remained as independent risk factors for incident CKD.

**Conclusions:**

A history of GDM/macrosomia or macrosomia alone, independent of subsequent diabetes mellitus was associated with significant risk for incident maternal CKD. Pregnancy may provide a unique situation to identify high-risk women at risk for CKD that could benefit from regular monitoring of kidney function and providing risk modifying strategies.

## Background

Chronic kidney disease (CKD) is an issue known as a considerable cause to increase morbidity and mortality with a prevalence of about 13.4% across the world [1]; the prevalence rate for CKD in Iran compared to the global rate is higher with a rate of 18.9% [2]. Moreover, we previously reported that more than 2% of the Iranian population develops CKD each year [3]. Impaired glucose tolerance, high blood pressure, and high body-mass index (BMI) have been associated with a higher risk of CKD development [4]. Tackling CKD and its risk factors could be deemed an obligation due to its insidious nature and significant burden on global health.

Gestational diabetes mellitus (GDM) is one of the most common complications of pregnancy that is a glucose intolerance status with onset or first diagnosis during pregnancy [5]. A global perspective study in 2016 reported that the Middle East and North Africa (MENA) region had the highest rank in the prevalence of GDM [6]. Results from a meta-analysis estimated the prevalence of GDM in Iran to be around 3.41% with a range of 1.3 to 18.6% [7]. GDM is increasing in prevalence among the Iranian population in tandem with the dramatic increase in the prevalence of obesity, physical inactivity, and glucose intolerance status including both prediabetes and type 2 diabetes mellitus (T2DM) [8–10]. Although most women with GDM will be normoglycemic after delivery [11], a history of GDM is known to increase the risk of subsequent T2DM, hypertension, metabolic syndrome, cardiovascular diseases (CVD), and endothelial dysfunction [12–18].

Macrosomia is defined as birth weight > 4000 g by the American College of Obstetricians and Gynecologists and has a prevalence of about 9% worldwide [19, 20]. It is reported that GDM and pre-GDM were associated with a higher prevalence of macrosomia [21] which is likely to increase the risk of future maternal T2DM [22, 23].

The question that whether GDM can independently increase the risk of CKD was poorly addressed in limited studies with inconsistent results [24–30]; most of them were based on a cross-sectional or retrospective design. A recent meta-analysis reported that no significant association between GDM and CKD was observed. However, black women with GDM had a higher risk of developing CKD [31]. Furthermore, to the best of our knowledge, there is no study investigating the impact of macrosomia on maternal CKD incidence.

Regarding the high burden of both GDM and CKD on the MENA region [4, 6] and relatively high prevalence of macrosomia [20], we aimed to study the relationship between macrosomia with or without GDM and incident CKD in the context of the oldest cohort of the region called the Tehran Lipid and Glucose Study (TLGS).

## Methods

The TLGS is a population-based longitudinal study carried out on individuals aged ≥3 years living in the urban area of metropolitan city of Tehran. This study aimed to determine the prevalence and incidence of non-communicable diseases and their related risk factors.

The TLGS recruitment was done in two phases (the first, 1999–2001 and the second, 2002–2005). Data collection is planned to continue for at least 20 years at approximately 3-year intervals (i.e. third phase: 2005–2008, fourth phase: 2009–2011, fifth phase: 2012–2015). The design and registration of the TLGS have been previously described [32].

### Study population

**Fig. 1:**
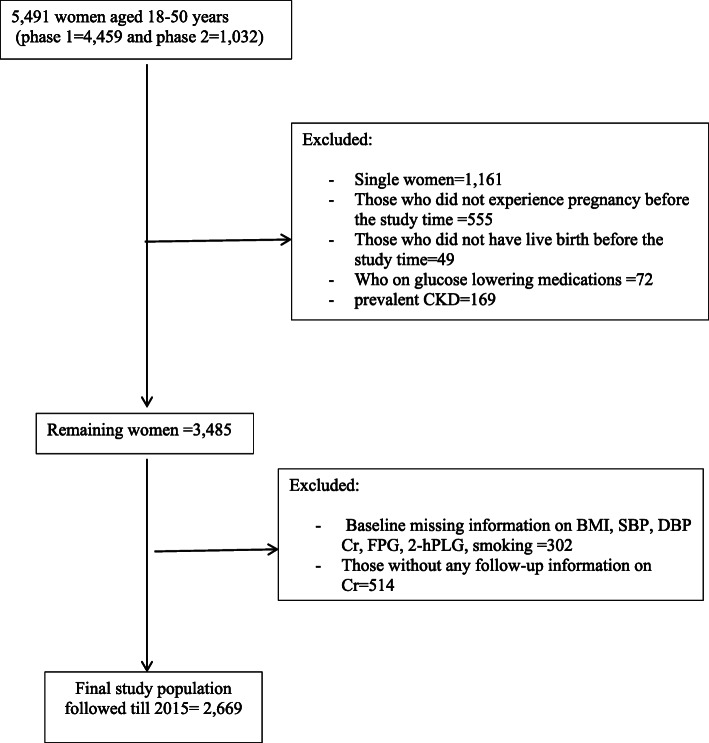
Flowchart of the study population. Abbreviations: CKD, chronic kidney disease; BMI, body mass index; SBP, systolic blood pressure; DBP, diastolic blood pressure; FPG, fasting plasma glucose; 2-hPLG, 2-h post load plasma glucose;Cr: creatinine

As shown in Fig. 1, of total 5,491 women aged 18–50 years (phase 1 = 4459 from; phase 2 = 1032), we excluded single women (*n* = 1161), women who did not experience pregnancy (*n* = 555), and had no live birth (*n* = 49) at the time of study recruitment. We also excluded individuals on glucose-lowering medication (*n* = 72) or prevalent CKD cases (*n* = 169). Of the remaining 3485 women, we also excluded participants with missing data for covariates including BMI, serum creatinine, fasting plasma glucose (FPG), 2-h post-load plasma glucose (2-hPLG), smoking status, systolic and diastolic blood pressures (SBP and DBP, respectively) considering the overlapped features (*n* = 302). Finally, after further exclusion of participants with missing follow-up data on serum creatinine (*n* = 514), our study sample included 2669 women who were followed until March 20, 2015. All participants signed informed written consent before being enrolled in the study. The study was reviewed and approved by the ethics committee of Shahid Beheshti University of Medical Sciences (Ethics approval reference number: IR.SBMU.MSP.REC.1399.175).

### Clinical, anthropometric, and laboratory measurements

Clinical, anthropometric, and laboratory data were collected according to the protocols of the TLGS [32]. Trained interviewers completed a detailed, pretested questionnaire for each participant. The questionnaire consisted of information regarding demographic data, marital status, education, smoking habits, medication history, family history of diabetes mellitus (FH-DM), and history of underlying comorbidities including hypertension, CKD, T2DM, GDM, and macrosomic baby delivery. Anthropometric variables consisted of weight, height, and waist circumference (WC). Qualified physicians measured height and weight in standing upright position with ordinary alignment of the shoulders while subjects were without shoes and dressed lightly using a tape meter and digital electronic weighing scale (Seca 707, Seca Corp., Hanover, MD, USA; range 0.1–150 Kg, recorded to the nearest 0.1 Kg), respectively. BMI was calculated by dividing body weight (Kg) by squared height (m^2^). WC was measured at the umbilical level.

The participants rested for 15 min in the sitting position to be prepared for blood pressure assessment. Skilled physicians measured blood pressure on two separate examinations with at least a 30 s interval from the right arm using a standardized mercury sphygmomanometer (calibrated by Iranian Institute of Standards and Industrial Researches). We regarded the average of the two measurements as the individual’s blood pressure.

A venous blood sample was collected from all participants between 7:00 and 9:00 am after 12–14 h of overnight fasting. The sample was then centrifuged within 30–45 min of collection. Oral glucose tolerance test was conducted using an 82.5 g glucose monohydrate solution (equivalent to 75 g anhydrous glucose) for all of the population not on glucose-lowering medication. Two hours after glucose ingestion a second blood sample is collected. FPG and 2-hPLG were assayed using enzymatic colorimetric glucose oxidase method; both intra- and inter-assay coefficient of variations (CVs) were less than 2.2%. Total cholesterol (TC) was assayed using the enzymatic colorimetric method with cholesterol esterase- cholesterol oxidase. For high density lipoprotein-cholesterol (HDL-C) measurement, precipitation of the apolipoprotein B containing lipoproteins with phosphotungstic acid was first done. For both TC and HDL-C, intra- and inter-assay CVs were 0.5 and 2% respectively. Serum creatinine levels were assayed by kinetic colorimetric Jaffe method. The sensitivity of the assay was 18 mmol/L (0.2 mg/dL) with measuring range of 18–1330 mmol/L (0.2–15 mg/dL). Reference intervals according to manufacturer’s recommendation were 80–115 mmol/L (0.9–1.3 mg/dL) and 53–97 mmol/L (0.6–1.1 mg/dL) in men and women, respectively. Both intra and inter-assay CVs were less than 3.1% in both baseline and follow-up phases. All biochemical assays were performed using commercial kits (Pars Azmoon Inc., Tehran, Iran) by a Selectra 2 auto-analyzer (Vital Scientific, Spankeren, The Netherlands). Assay performance was monitored after every 25 tests using lyophilized serum controls in normal and pathologic ranges and all samples were analyzed when internal quality control met the standard acceptance criteria.

### Definition of terms

According to the Kidney Disease Outcome Quality Initiative guidelines, CKD is defined as kidney damage or glomerular filtration rate (GFR) < 60 ml/min/1.73 m^2^ present for more than 3 months which translates to CKD stage 3–5 [33]. For this study, estimated GFR (eGFR) was calculated using a commonly abbreviated prediction equation, provided by Chronic Kidney Disease Epidemiology Collaboration (CKD-EPI) study as follows:

EPI: eGFR = 141 × min (S_cr_/κ, 1) ^α^ × max (S_cr_/κ, 1) ^− 1.209^ × 0.993^age^ × 1.018 (if female) × 1.159 (if black).

In this equation, serum creatinine (S_cr_) was measured in mg/dL and age in years. κ is 0.7 and 0.9 for men and women, respectively, α is − 0.329 and − 0.411 for men and women; min indicates the minimum S_cr_/κ or 1, and max indicates the maximum S_cr_/κ or 1. Estimated GFR is also expressed as mL/min/1.73 m^2^ [34].

A positive FH-DM was considered if any first-degree relatives of the participant had T2DM. Parity was defined as pregnancies longer than 20 weeks leading to either live birth or stillbirth. Hypertension was defined as either taking anti-hypertensive drug treatment or SBP > 140 mmHg or DBP > 90 mmHg. A person with a BMI of 30 Kg/m^2^ or more was considered obese. According to the first report of the Iranian National Committee of Obesity central adiposity was defined as WC ≥ 90 cm for both men and women [35]. Hypercholesterolemia was defined as TC≥5.18 mmol/L or using lipid-lowering medications. Low HDL-C was defined as HDL-C < 1.036 mmol/L for men and < 1.295 mmol/L for women. Participants were stratified either into married or widowed/divorced groups based on their marital status. Smoking habits were categorized into former, current, and non smokers. A current smoker was considered a person who smokes cigarettes, pipe, or hookah daily or occasionally; those who didn’t currently smoke with a previous history of smoking were defined as former smokers, and those without any history of smoking were categorized as never smoker (as the reference group). Educational level was categorized into 3 groups of < 6 years, 6–12 years, and > 12 years of education.

### Definition of exposure groups

In the current study, we categorized the study population into three groups: group 1; GDM/macrosomia, including participants with a self-reported history of macrosomia (birth weight> 4000 g) or GDM and without T2DM, group 2; incident cases of newly diagnosed T2DM (NDM), consisted of participants who had FPG ≥ 7 mmol/L or 2-hPLG ≥11.1 mmol/L in the presence or abcence of GDM/Macrosomia, and, group 3; the reference group including women without prior history of GDM/macrosomia and free of NDM (no GDM/macrosomia and no NDM).

### Statistical analysis

Continuous and categorical variables were reported as mean (standard deviation-SD) or frequencies (%) as appropriate. Comparison of baseline characteristics of the three groups of participants including women with a history of GDM/macrosomia, women with incident NDM, and women without a history of GDM/macrosomia and NDM (reference group) was performed using the ANOVA test for continuous variables and Chi-square test for categorical variables. The crude incidence rate (95% confidence interval- CI) was calculated by dividing the number of new cases of CKD by person-years at risk. Cox proportional hazard regression was used to examine the relationship between GDM/macrosomia and incident NDM categories with incident CKD in 3 models; model 1, unadjusted; model 2, adjusted only for baseline age and model 3, adjusted for baseline age, and other potential confounders according to our previous study [3] including BMI, WC, number of parities, smoking status, educational level, gestational hypertension, eGFR, SBP, DBP, anti-hypertensive medication, and FH-DM.

A Kaplan-Meier survival curve was used to estimate the cumulative incidence of CKD. To show the robustness of our findings, we tested the interaction between the different status of general obesity, central adiposity, hypercholesterolemia, lowHDL-C, and hypertension with our main exposures (i.e. GDM/macrosomia and NDM) with incident CKD in age-adjusted analysis, using the likelihood ratio test [4]. Schoenfeld’s global test of residuals was used to check the proportionality assumption of the multivariable Cox regression and no interference was observed.

The level of significance was set at < 0.05 for all statistical analyses. All statistical analyses were done using STATA statistical package version 14 SE (StataCorp, TX, USA).

## Results

Of all 2669 participants, 204 women (7.64%) had a history of GDM/macrosomia, 113 subjects (4.23%) were incident cases of NDM and 2352 participants (88.13%) were neither with T2DM nor had a history of GDM/macrosomia. The baseline characteristics of these three groups are described in Table 1. The mean age (SD) and BMI of all participants at baseline were 36.2 (7.3) years and 27.7 (4.6) Kg/m^2^, respectively. Generally, incident cases of NDM showed a less favorable situation for all baseline characteristics compared to other groups, excluding incident CKD and obesity, which were more prevalent in the GDM/macrosomia group (all *P* values < 0.05). We observed no significant difference in smoking status and marital status between study groups. Moreover, the frequency of incident CKD cases among GDM/macrosomia were higher compared with incident cases of NDM and T2DM-free individuals (33.8 vs. 30.1 vs. 21.7%, respectively).

**Table 1:** Baseline characteristics of the participants based on different categories of GDM/macrosomia, with and without diabetes

Variables	Total (***n*** = 2669)	No diabetes (*n* = 2352)	GDM/macrosomia (*n* = 204)	NDM (*n* = 113)	***P*** value
Age, years	36.2 (7.3)	35.6 (7.2)	39.5 (6.5)	42.5 (5.3)	< 0.001
BMI (Kg/m^2^)	27.7 (4.6)	27.4 (4.5)	30.1 (4.5)	30.3 (4.9)	< 0.001
WC (cm)	87 (11.5)	86 (11.1)	93.6 (11.1)	95.8 (11.9)	< 0.001
SBP (mmHg)	112.2 (13.7)	111.3 (13.2)	114.4 (14.1)	125.3 (14.9)	< 0.001
DBP (mmHg)	75.7 (9.7)	75.3 (9.5)	77.3 (10.1)	82.4 (9.4)	< 0.001
FPG (mmol/L)	4.99 (0.80)	4.88 (0.49)	5.06 (0.56)	7.21 (2.04)	< 0.001
2-hPLG (mmol/L)	6.29 (2.37)	5.90 (1.45)	6.33 (1.56)	14.4 (3.94)	< 0.001
eGFR (ml/min/1.73 m^2^)	78.8 (10.8)	79.3 (10.9)	75.7 (9.7)	74.4 (8.6)	< 0.001
TC (mmol/L)	5.15 (1.03)	5.10 (1.03)	5.29 (0.94)	5.74 (1.07)	< 0.001
HDL-C (mmol/L)	1.14 (0.29)	1.15 (0.29)	1.10 (0.28)	1.04 (0.25)	< 0.001
Parity	2.6 (1.4)	2.5 (1.3)	3.2 (1.4)	3.6 (1.5)	< 0.001
Marital status, n (%)					0.658
- Married	2569 (96.3)	2226 (96.3)	196 (96.1)	107 (94.7)	
- Widow/divorced	100 (3.7)	86 (3.7)	8 (3.9)	6 (5.3)	
Smoking status, n (%)					0.62
- Never	2530 (94.8)	2232 (94.9)	192 (94.1)	106 (93.8)	
- Former	21 (0.8)	20 (0.9)	1 (0.5)	0 (0)	
- Current	118 (4.4)	100 (4.3)	11 (5.4)	7 (6.2)	
Education, n (%)					< 0.001
- < 6 years	629 (23.6)	516 (21.9)	61 (29.9)	52 (46)	
- 6–12 years	1786 (66.9)	1597 (67.9)	132 (64.7)	57 (50.4)	
- ≥ 12 years	254 (9.5)	239 (10.2)	11 (5.4)	4 (3.5)	
Obesity, n (%)	750 (28.1)	596 (25.3)	101 (49.5)	53 (46.9)	< 0.001
Hypertension, n (%)	292 (10.9)	234 (9.9)	27 (13.2)	31 (27.4)	< 0.001
FH-DM, n (%)	769 (28.8)	655 (27.8)	71 (34.8)	43 (38.1)	0.009
Anti-hypertensive medication, n (%)	69 (2.59)	56 (2.38)	5 (2.45)	8 (7.08)	0.009
Lipid-lowering medication, n (%)	34 (1.27)	25 (1.06)	5 (2.45)	4 (3.54)	0.021
Incident CKD, n (%)	613 (23.0)	510 (21.7)	69 (33.8)	34 (30.1)	< 0.001

Abbreviations: NDM: newly diagnosed diabetes; GDM, gestational diabetes mellitus; BMI, body mass index; WC, waist circumference; SBP, systolic blood pressure; DBP, diastolic blood pressure; FPG, fasting plasma glucose; 2-hPLG, 2-h post load plasma glucose; eGFR, estimated glomerular filtration rate; TC, total cholesterol; HDL-C, high density lipoprotein-cholesterol; FH-DM, family history of diabetes mellitus; CKD, chronic kidney disease

Values are shown as Mean (SD) and number (%) for continuous and categorical variables, respectively

**Fig. 2:**
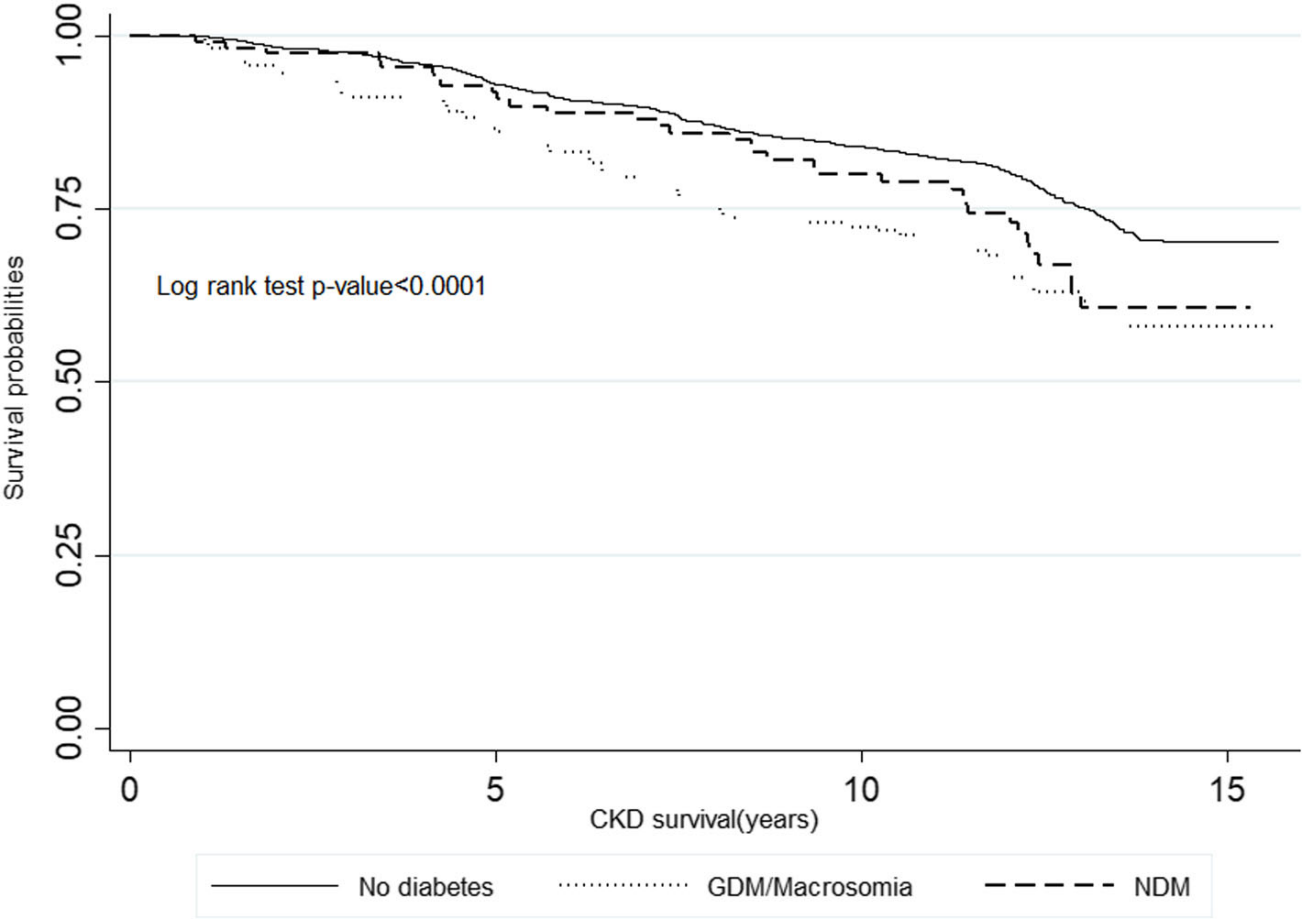
Kaplan-Meier survival curve for cumulative incident CKD of different categories of GDM/macrosomia, with and without diabetes. Abbreviations: CKD, chronic kidney disease; GDM, gestational diabetes mellitus; NDM: newly diagnosed type 2 diabetes mellitus

During a median follow-up (interquartile range) of 11.9 years (8.3–13.2), 613 incident cases of CKD were identified with the corresponding incidence rate of 21.6 (95% CI: 20–23.4) per 1000 person-year. Figure 2 shows the Kaplan-Meier survival curve for the cumulative CKD incidence in the three study groups (log-rank test *P*-value < 0.001).

**Fig. 3:**
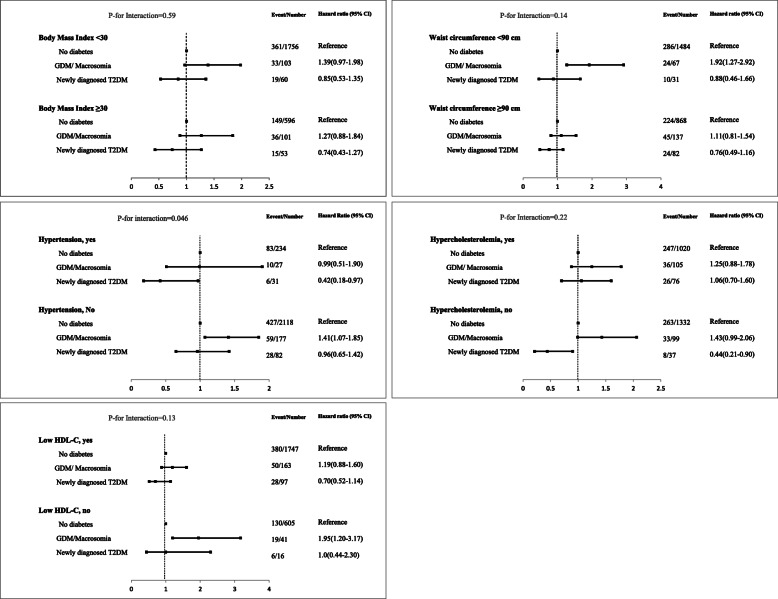
Relationship between GDM/macrosomia and NDM with incident CKD stratified by body mass index, waist circumference, blood pressure status, hypercholesterolemia, and low HDL-C. Hazard ratios and interaction term was calculated in the age-adjusted analysis. Abbreviations: CKD, chronic kidney disease; GDM, gestational diabetes mellitus; T2DM, type 2 diabetes mellitus; HDL-C: high-density lipoprotein cholesterol; NDM: newly diagnosed diabetes

Crud, age and multivariable adjusted hazard ratio (HR) of GDM/macrosomia and incident NDM with incident CKD is shown in Table 2. Accordingly, only GDM/macrosomia showed 33% significant risk for incident CKD in the age-adjusted analysis [HR: 1.33 (95% CI: 1.03–1.71)]; the risk still remained significant after further adjustment for potential risk factors [HR: 1.32 (95% CI: 1.02–1.72), *P* = 0.032]. In the multivariable analysis, aging [HR: 1.06 (95% CI: 1.04–1.08)], current smoker [HR: 1.42 (95% CI: 1.00–1.99)], level of eGFR [HR: 0.91 (95% CI: 0.90–0.92)] and SBP [HR: 1.01 (95% CI: 1.00–1.02)] also remained independent risk factors. The results remained essentially unchanged when we replaced the definition of GDM/macrosomia with a history of macrosomia alone as shown in Table 3. Accordingly, having a history of macrosomia was associated with a higher risk for incident CKD of 36% [HR: 1.36 (95% CI: 1.04–1.78)]. However, when we replaced the definition of GDM/macrosomia with a history of GDM alone no association was found between GDM and incident CKD [HR: 0.92 (95% CI: 0.34–2.46)] (Table 4). In stratified analyses as shown in Fig. 3, we did not find a significant interaction between different study groups and general obesity (*P* = 0.59), central adiposity (*P* = 0.14), hypercholesterolemia (*P* = 0.22), and low HDL-C (*P* = 0.13). However, the data analysis showed a significant interaction between hypertension status and different study groups (*P* = 0.046). Hence, only among the non-hypertensive population, GDM/macrosomia group showed a significant risk for incident CKD [HR: 1.41 (95% CI: 1.07–1.85)]. Moreover, unexpectedly we found that the incident NDM group among the hypertensive population had a lower risk for CKD; however, considering only 6 events in this group, the effect size is unstable [HR: 0.42 (95% CI: 0.18–0.97)].

**Table 2:** Hazard ratios of different categories of GDM/macrosomia, with and without diabetes for incident CKD

Variables	Model 1		Model 2		Model 3	
	HR (95% CI)	***P*** value	HR (95% CI)	***P*** value	HR (95% CI)	***P*** value
Study groups						
- No diabetes	Reference		Reference		Reference	
- GDM/macrosomia	1.78(1.39–2.30)	< 0.001	1.33 (1.03–1.71)	0.029	1.32 (1.02–1.72)	0.032
- NDM	1.45(1.02–2.05)	0.037	0.80 (0.56–1.39)	0.22	0.74 (0.52–1.07)	0.109
Age, years			1.10(1.09–1.11)	< 0.001	1.06 (1.04–1.08)	< 0.001
BMI (Kg/m^2^)	–	–	–	–	1.01 (0.98–1.05)	0.43
WC (cm)	–	–	–	–	0.99 (0.98–1.00)	0.38
Parity	–	–	–	–	0.98 (0.92–1.06)	0.68
Smoking status						
- Never	–	–	–	–	Reference	
- Former					0.87 (0.36–2.10)	0.75
- Current smoker	–	–	–	–	1.42 (1.00–1.99)	0.048
Education						
- < 6 years	–	–	–	–	Reference	
- 6–12 years	–	–	–	–	1.20 (0.98–1.47)	0.077
- ≥ 12 years	–	–	–	–	1.18 (0.84–1.66)	0.34
Gestational hypertension	–	–	–	–	0.97 (0.92–1.03)	0.37
eGFR (ml/min/1.73 m^2^)	–	–	–	–	0.91 (0.90–0.92)	< 0.001
SBP (mmHg)	–	–	–	–	1.01 (1.00–1.02)	0.043
DBP (mmHg)	–	–	–	–	0.99 (0.98–1.005)	0.26
Anti-hypertensive medication	–	–	–	–	0.99 (0.63–1.53)	0.95
FH-DM	–	–	–	–	1.01 (0.85–1.20)	0.88

Model 1: Study groups (unadjusted model)

Model 2: adjusted for baseline age

Model 3: adjusted for baseline age, BMI, WC, number of parities, smoking status, educational level, gestational hypertension, eGFR, SBP, DBP, anti-hypertensive medication, and FH-DM

Abbreviations: NDM: Newly diagnosed  type 2 diabetes mellitus; GDM, gestational diabetes mellitus; CKD, chronic kidney disease; HR, hazard ratio; CI, confidence interval; BMI, body mass index; WC, waist circumference; eGFR, estimated glomerular filtration rate; SBP, systolic blood pressure; DBP, diastolic blood pressure; FH-DM, family history of diabetes mellitus

**Table 3:** Hazard ratios of different categories of macrosomia, with and without diabetes for incident CKD

	Model 1		Model 2		Model 3	
	HR (95% CI)	***P*** value	HR (95% CI)	***P*** value	HR (95% CI)	***P*** value
Study groups (n)						
- No diabetes (2380)	Reference		Reference		Reference	
- Macrosomia (176)	2.0(1.54–2.59)	< 0.001	1.37 (1.06–1.78)	0.017	1.36 (1.04–1.78)	0.023
- NDM (113)	1.45(1.03–2.05)	0.035	0.80 (0.56–1.43)	0.22	0.74 (0.52–1.07)	0.111
Age, years	–	–	1.10(1.09–1.11)	< 0.001	1.06 (1.04–1.08)	< 0.001
BMI (Kg/m^2^)	–	–	–	–	1.01 (0.98–1.05)	0.43
WC (cm)					0.99 (0.98–1.01)	0.38
Parity					0.98 (0.92–1.06)	0.68
Smoking status						
- Never	–	–	–	–	Reference	
- Former					0.87 (0.36–2.10)	0.75
- Current smoker	–	–	–	–	1.42 (1.00–2.00)	< 0.001
Education						
- < 6 years	–	–	–	–	Reference	
- 6–12 years	–	–	–	–	1.20 (0.98–1.47)	0.078
- ≥ 12 years	–	–	–	–	1.18 (0.84–1.66)	0.34
Gestational hypertension	–	–	–	–	0.97 (0.92–1.03)	0.37
eGFR (ml/min/1.73 m^2^)	–	–	–	–	0.91 (0.90–0.92)	< 0.001
SBP (mmHg)	–	–	–	–	1.01 (1.00–1.02)	0.042
DBP (mmHg)	–	–	–	–	0.99 (0.98–1.01)	0.26
Anti-hypertensive medication	–	–	–	–	0.99 (0.64–1.53)	0.96
FH-DM	–	–	–	–	1.02 (0.85–1.21)	0.90

Model 1: Study groups (unadjusted model)

Model 2: adjusted for baseline age

Model 3: adjusted for baseline age, BMI, WC, number of parities, smoking status, educational level, gestational hypertension, eGFR, SBP, DBP, anti-hypertensive medication, and FH-DM

Abbreviations: NDM: newly diagnosed type 2 diabetes mellitus; CKD, chronic kidney disease; HR, hazard ratio; CI, confidence interval; n, number; BMI, body mass index; WC, waist circumference; eGFR, estimated glomerular filtration rate; SBP, systolic blood pressure; DBP, diastolic blood pressure; FH-DM, family history of diabetes mellitus

**Table 4:** Hazard ratios basof different categories of GDM, with and without diabetes for incident CKD

	Model 1		Model 2		Model 3	
	HR (95% CI)	***P*** value	HR (95% CI)	***P*** value	HR (95% CI)	***P*** value
Study groups (n)						
- No diabetes (2527)	Reference		Reference		Reference	
- Macrosomia (29)	0.61(0.23–1.66)	0.34	0.83(0.31–2.21)	0.71	0.92(0.34–2.46)	0.86
- NDM (113)	1.36(0.96–1.93)	0.08	0.77(0.54–1.10)	0.15	0.71(0.50–1.02)	0.06
Age, years	–	–	1.10(1.09–1.12)	< 0.001	1.06(1.04–1.08)	< 0.001
BMI (Kg/m^2^)	–	–	–	–	1.01(0.98–1.05)	0.4
WC (cm)					0.99(0.98–1.01)	0.48
Parity					0.99(0.92–1.06)	0.77
Smoking status						
- Never	–	–	–	–	Reference	
- Former					0.85(0.35–2.05)	0.72
- Current smoker	–	–	–	–	1.40(0.99–2.0)	0.05
Education						
- < 6 years	–	–	–	–	Reference	
- 6–12 years	–	–	–	–	1.20(0.98–1.47)	0.08
- ≥ 12 years	–	–	–	–	1.18(0.84–1.66)	0.34
Gestational hypertension	–	–	–	–	0.97(0.92–1.03)	0.3
eGFR (ml/min/1.73 m^2^)	–	–	–	–	0.91(0.90–0.92)	< 0.001
SBP (mmHg)	–	–	–	–	1.01(1.00–1.02)	0.039
DBP (mmHg)	–	–	–	–	0.99(0.98–1.01)	0.24
Anti-hypertensive medication	–	–	–	–	0.97(0.63–1.51)	0.9
FH-DM	–	–	–	–	1.01(0.85–1.21)	0.87

Model 1: Study groups (unadjusted model)

Model 2: adjusted for baseline age

Model 3: adjusted for baseline age, BMI, WC, number of parities, smoking status, educational level, gestational hypertension, eGFR, SBP, DBP, anti-hypertensive medication, and FH-DM

Abbreviations: NDM: newly diagnosed type 2 diabetes mellitus; GDM: gestational diabetes mellitus; CKD, chronic kidney disease; HR, hazard ratio; CI, confidence interval; n, number; BMI, body mass index; WC, waist circumference; eGFR, estimated glomerular filtration rate; SBP, systolic blood pressure; DBP, diastolic blood pressure; FH-DM, family history of diabetes mellitus

As a sensitivity analysis, the inverse probability weighting of the multinomial propensity score [36] was used to balance characteristics among three exposure groups (i.e. those without GDM/macrosomia and diabetes, with GDM/Macrosomia, and with undiagnosed diabetes). The analysis adjusted for baseline age, BMI, WC, number of parities, smoking status, educational level, gestational hypertension, eGFR, SBP, DBP, anti-hypertensive medication, and FH-DM. The result remained unchanged, the HRs (95% CI) for GDM/macrosomia and NDM groups were [HR: 1.34 (95% CI: 1.03–1.74)] and 1.05 (95% CI: 0.75–1.50)], respectively (data not shown).

## Discussion

For more than a decade of follow-ups among women aged 18–50 years in Tehran, we found that a history of GDM/macrosomia was associated with more than 30% higher risk of CKD, independent of a large set of important potential confounders including age, general obesity, central adiposity, parity, educational level, gestational hypertension, smoking status, FH-DM, SBP, DBP, using anti-hypertensive medication, and eGFR. This correlation also applied to women with a history of macrosomia alone. Moreover, we also found that in women with a history of GDM/macrosomia, non-hypertensive participants had a higher risk of CKD incidence compared to hypertensive participants.

The results of other studies examining the relationship between GDM and CKD are incompatible for comparison with each other, due to the different approaches applied for diagnosis of GDM, different sample sizes, various study designs (cross-sectional, retrospective, or prospective), the selection method of participants (hospital or population-based study), and number and type of confounders. Moreover, all of the studies in this field were confined to the United States (US), European and Israeli women, and no population-based study examined this relationship in the MENA region [24–30].

The question regarding the relationship between GDM and subsequent impaired kidney function was first addressed by Friedman et al. [24] in a cross-sectional study of 72 Israeli women with a history of GDM, and as a result, a higher risk of microalbuminuria was observed. In another cross-sectional study with larger sample size, US women with GDM alone had similar odds of CKD stages 1–2 (odds ratio: 1.54) comparable with women with overt diabetes (odds ratio: 1.68) [26]. These findings were supported by another retrospective hospital-based cohort study in Israel which observed that GDM was a significant risk factor for long-term renal morbidity [27]. To the best of our knowledge, only 3 prospective cohorts have examined the relationship between GDM and incident CKD. The first study was conducted in 2015 with a sample size of 320 Canadian women of whom 100 cases had a history of GDM, confirmed by oral glucose tolerance test, and after 3 years of follow-up, they reported that current glucose intolerance, but not GDM, was associated with microalbuminuria. Importantly, in contrast to previous studies, the authors considered current glucose intolerance and eGFR as important confounders in their data analysis [28]. In the Coronary Artery Risk Development in Young Adults (CARDIA) Study, the presence of the self-reported GDM among black women, was associated with incident CKD, mainly attributable to albuminuria. This association did not change after adjustment for a large set of pre-pregnancy risk factors for both GDM and CKD including eGFR and fasting glucose concentrations [30]. In a prospective study of 607 Danish women with GDM with a mean follow-up of 13 years, a higher rate of increased eGFR levels was seen compared to the control group, indicating an early stage of renal damage. Moreover, the authors also showed that renal damage defined as increased urine albumin-creatinine ratio (UACR), was limited to GDM cases with subsequent development of T2DM [29]. In a recent meta-analysis including 2 studies, it was shown that history of GDM was associated with a 20% increased risk of CKD, the values which did not reach the significance level [risk ratio: 1.19 (95% CI: 0.72–1.97), I^2^: 66%] [31].

Interestingly, in the current study, we showed for the first time that a history of macrosomia alone in the absence of a history of self-reported GDM was also significantly associated with incident CKD. The association between the history of macrosomia and the development of T2DM and CVD had been previously reported [22, 23, 37]. Studies have also shown a strong association between pre-pregnancy maternal obesity, decreased insulin sensitivity, and increased insulin resistance with the delivery of macrosomic babies [38, 39]. Hence, the significant relationship between the history of macrosomia and incident CKD among Iranian women might be attributable to the high prevalence of pre-pregnancy maternal obesity and metabolic syndrome, two of the main risk factors for incident CKD, among Iranian women [4, 10, 40].

Although the precise mechanism underlying the higher risk of CKD among GDM cases observed in some studies is unknown, several potential mechanisms may explain the relationship. Interleukin-6, C-reactive protein, and plasminogen activator inhibitor-1 which are inflammatory factors that can anticipate future CVD and CKD [41–44] are significantly higher in women with a history of GDM compared to those with no prior history [45, 46]. Moreover, a lower plasma adiponectin concentration, an anti-inflammatory factor, has been observed in women with GDM history [47]. Vascular endothelial dysfunction is associated with incident CKD [48], which is more prevalent in GDM women compared to the control group, in terms of increased wall stiffness [49], increased peripheral vascular resistance [46], impaired endothelium-dependent vasodilatation [50], and impaired acetylcholine-induced skin vasodilatation [49, 51]. Subclinical inflammation and vascular endothelial dysfunction in GDM, in the absence of overt T2DM, may suggest a possible link between GDM and subsequent CKD.

The strength of this study lies in the long-term follow-up of participants in a population-based study with a high burden of CVD risk factors and CKD [2, 52]. Moreover, the higher risk of CKD was also shown in the presence of well-known CKD risk factors including age, smoking status, eGFR, and SBP [4]. Importantly we also considered incident T2DM as another important confounder in our data analysis. Furthermore, most confounders were precisely measured using standardized protocols rather than relying on self-report data. We also for the first time showed the significant relationship between the history of macrosomia and incident CKD.

Our study has important limitations that should be considered. First, GDM was defined based on self-reported data; an approach applied in multiple other studies [30]. Second, we defined CKD according to a single estimate of eGFR and accepted the possibility of overestimation of CKD cases. Estimated GFR shows intra-individual biological variability and preferably needs a second creatinine measurement to accurately represent kidney function. The use of successive eGFR, had they been accessible, would have most likely reduced the incidence of CKD stage 3–4 but might not have affected the relationship between the 3 study groups (GDM/macrosomia, incident NDM, and reference group) and CKD. Despite this, most of the population-based studies focusing on CKD as the outcome used single serum creatinine measurements [29]. Furthermore, a recently published systematic review and meta-analyses aimed to assess the predictive role of obesity, overweight, and BMI on the risk of new-onset CKD, showed the association between obesity and low eGFR did not affect by the number of eGFR measurements [53]. Third, we did not validate the CKD-EPI equation in a local population, and this could also lead to an overestimation of the incidence of CKD. Fourth, the definition of CKD was built only upon eGFR criteria, since no valid data for urine protein excretion were available in the TLGS. Fifth, this study was conducted in the metropolitan city of Tehran, hence other studies particularly in the rural area should examine this association.

## Conclusions

We concluded that Iranian women with a history of GDM/macrosomia or even macrosomia alone are at higher risk for subsequent CKD, independent of traditional CKD risk factors as well as incident T2DM. Given the high incidence of CKD among the Iranian population [3], pregnancy may provide a unique situation to identify high-risk women at risk for CKD that could benefit from regular monitoring of kidney function and providing risk modifying strategies.

## Data Availability

The data supporting this study is available through the corresponding author upon reasonable request.

## Abbreviations

TLGS: Tehran Lipid and Glucose Study
CARDIA: Coronary Artery Risk Development in Young Adults
MENA: Middle East and North Africa
BMI: Body mass index
TC: Total cholesterol
WC: Waist circumference
SBP: Systolic blood pressure
DBP: Diastolic blood pressure
HDL-C: High-density lipoprotein cholesterol
2-hPLG: 2-h post-load plasma glucose
FPG: Fasting plasma glucose
Cr: Creatinine
GFR: Glomerular filtration rate
eGFR: estimated glomerular filtration rate
T2DM: Type 2 diabetes mellitus
NDM: Newly diagnosed type 2 diabetes mellitus
CKD: Chronic kidney disease
CVD: Cardiovascular diseases
GDM: Gestational diabetes mellitus
FH-DM: Family history of diabetes mellitus
CI: Confidence interval
CV: Coefficient of variations
HR: Hazard ratio
IQR: Interquartile Range
SD: Standard deviation
